# Mass Spectrometry-Based Redox and Protein Profiling of Failing Human Hearts

**DOI:** 10.3390/ijms22041787

**Published:** 2021-02-11

**Authors:** Tamara Tomin, Matthias Schittmayer, Simon Sedej, Heiko Bugger, Johannes Gollmer, Sophie Honeder, Barbara Darnhofer, Laura Liesinger, Andreas Zuckermann, Peter P. Rainer, Ruth Birner-Gruenberger

**Affiliations:** 1 Faculty of Technical Chemistry, Institute of Chemical Technologies and Analytics, Vienna University of Technology-TU Wien, Getreidemarkt 9/164, 1060 Vienna, Austria; tamara.tomin@tuwien.ac.at; 2 Diagnostic and Research Institute of Pathology, Medical University of Graz, Stiftingtalstrasse 6, 8010 Graz, Austria; sophie.honeder@medunigraz.at (S.H.); b.darnhofer@medunigraz.at (B.D.); laura.liesinger@medunigraz.at (L.L.); 3 BiotechMed-Graz, Mozartgasse 12/II, 8010 Graz, Austria; simon.sedej@medunigraz.at; 4 Division of Cardiology, Medical University of Graz, Auenbruggerplatz 15, 8036 Graz, Austria; heiko.bugger@medunigraz.at (H.B.); johannes.gollmer@medunigraz.at (J.G.); 5 Faculty of Medicine, University of Maribor, 2000 Maribor, Slovenia; 6 Cardiac Transplantation, Department of Cardiac Surgery, Medical University of Vienna, Spitalgasse 23, 1090 Vienna, Austria; andreas.zuckermann@meduniwien.ac.at

**Keywords:** failing hearts, cardiomyopathy, oxidative stress, redox proteomics

## Abstract

Oxidative stress contributes to detrimental functional decline of the myocardium, leading to the impairment of the antioxidative defense, dysregulation of redox signaling, and protein damage. In order to precisely dissect the changes of the myocardial redox state correlated with oxidative stress and heart failure, we subjected left-ventricular tissue specimens collected from control or failing human hearts to comprehensive mass spectrometry-based redox and quantitative proteomics, as well as glutathione status analyses. As a result, we report that failing hearts have lower glutathione to glutathione disulfide ratios and increased oxidation of a number of different proteins, including constituents of the contractile machinery as well as glycolytic enzymes. Furthermore, quantitative proteomics of failing hearts revealed a higher abundance of proteins responsible for extracellular matrix remodeling and reduced abundance of several ion transporters, corroborating contractile impairment. Similar effects were recapitulated by an in vitro cell culture model under a controlled oxygen atmosphere. Together, this study provides to our knowledge the most comprehensive report integrating analyses of protein abundance and global and peptide-level redox state in end-stage failing human hearts as well as oxygen-dependent redox and global proteome profiles of cultured human cardiomyocytes.

## 1. Introduction

Oxidative stress plays a significant role in the development and progression of various pathologies [1]. In heart disease, oxidative stress is associated with risk factors such as obesity, diabetes, and aging, and contributes to the pathogenesis of atherosclerosis, ischemic heart disease, and ultimately heart failure (HF) [2,3,4,5]. Both in ischemic (ICM) and dilative (DCM) cardiomyopathies, oxidative stress is crucially involved in disease development and progression [2,6] and is associated with the severity of symptoms [2,7]. Preclinical work led to promising results regarding potential redox-based therapies for cardiac pathologies; however, the translation to clinical practice is still hampered by several factors [2,6]. First, the redox balance is a highly dynamic process and, thus, rapid and reliable determination of oxidation is required, which is often difficult to achieve in the clinical setting. Second, global redox levels do not depict the oxidation level of individual peptides, which may both convey prognostic information and impact protein functionality. Lastly, we still lack consistent read-outs in non-invasive and easily obtainable biomaterial sources that correlate with tissue redox states.

One of the ways to address oxidative-stress-induced adaptations of the cardiac proteome is to carry out mass-spectrometry (MS)-based proteomics. In recent years, MS-based proteomics has gained special attention in the field of cardiovascular research, aiding biomarker discoveries and providing insight into novel metabolic pathways [8]. In this regard, proteomics investigations on both patient [9] and animal models [10,11] of failing heart have been carried out, providing much needed information toward a deeper understanding of the pathophysiology of HF. Furthermore, the importance of oxidative post-translational modifications (oxPTMs) is also increasingly recognized, particularly in the field of cardiovascular research. oxPTMs can lead to deregulation of signaling, can compromise cellular homeostasis, and contribute to disease progression [12,13]. Although analysis of oxPTMs can be challenging, often requiring additional derivatisation steps [12,13], new developments in the MS field enable sensitive and precise measurements of even the most technically intricate oxidative modifications [14,15,16]. 

In this study, we applied a novel, comprehensive mass-spectrometry approach to precisely characterize the redox state and decipher global as well as peptide-specific reactive oxygen and nitrogen species (RNOS) signaling in failing human hearts. To that end, we carried out an unbiased redox and quantitative proteomics characterization of a sizable number of end-stage failing human left-ventricular (LV) tissue samples (*n* = 15 in total; end-stage DCM and ICM, *n* = 3 and 12, respectively), which were compared to 10 nonfailing human-control LV specimens. All tissue biopsies were preserved from artificial oxidation that could occur during sample preparation and were analysed by a two-step alkylation approach [17], providing information about one of the most abundant endogenous antioxidants, glutathione (GSH) and glutathione disulfide (GSSG) [18], as well as the redox status of individual redox-sensitive (cysteine-containing) peptides. In addition, we report quantitative changes in protein abundances correlated with failing heart. Compared to other methodologies, our combined approach provided us great benefit of a reliable integrative analysis, as all investigations were carried out on the same tissue explants and always measured by means of mass spectrometry. As a result, we present to our knowledge the most comprehensive characterization of redox state and proteome profile of failing human hearts. In addition, many of the reported changes could also be recapitulated in a human cardiomyocyte line (AC16) cultured under a different oxygen concentration to induce oxidative stress (normoxia (21%) versus physioxia (5%)).

## 2. Results

### 2.1. The GSH/GSSG Ratio is Reduced in Failing Hearts

**Figure 1:**
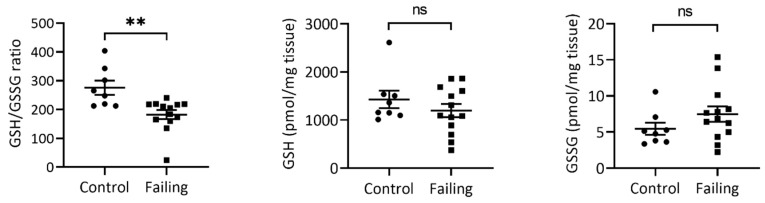
Failing hearts display a significantly reduced GSH/GSSG ratio (**left**). Absolute GSH levels (**middle**) in failing hearts are slightly reduced (not significant, *p* = 0.313), while GSSG content (**right**) is slightly increased in failing (not significant, *p* = 0.194). Values represent mean values and S.E.M. *n* (control) = 10, *n* (Failing hearts) = 15. ** Student *t*-test *p*-value < 0.01; ns—not significant.

Glutathione status as a proxy for oxidative stress has been proposed as a prognostic marker for atherosclerosis, coronary disease, and heart failure [4,19,20]. Therefore, we first applied our recently published two-step alkylation approach to measure the GSH/GSSG ratio [17] of 15 failing LV tissue samples (failing heart due to ICM or DCM) compared to nonfailing control hearts (*n* = 10). As expected, failing hearts showed significantly a reduced GSH/GSSG ratio compared to controls (Figure 1, left panel), which was due to a slightly lower GSH (Figure 1, mid panel, not significant) and slightly higher GSSG content (Figure 1, right panel, not significant) in each of the failing heart samples, indicating higher oxidative-stress exposure of failing myocardium.

### 2.2. Redox Proteomics Reveal Higher Oxidation of Glucose Metabolic Enzymes and Proteins of the Contractile Machinery

**Figure 2:**
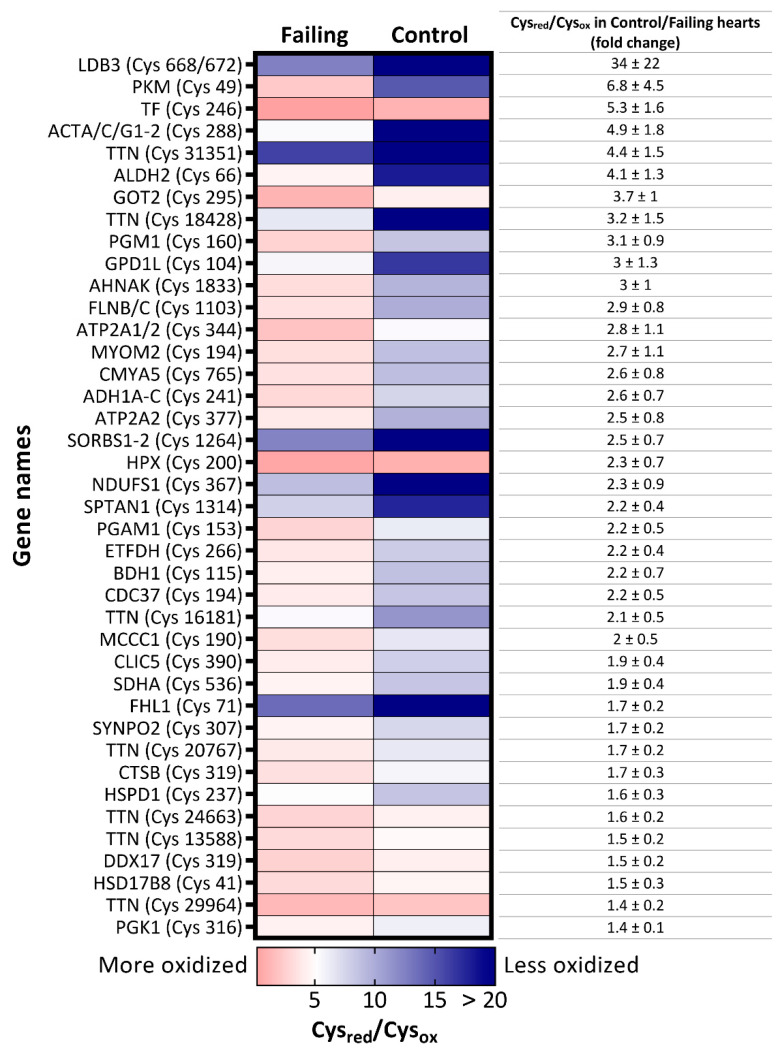
Heatmap of significantly more oxidized-cysteine residues in failing hearts. Significantly more oxidized cysteine-containing peptides *p*-value < 0.05, fold-change (Cys_red_/Cys_ox_ ratio of control versus failing heart >1.35) in failing hearts are labeled with gene names of their corresponding proteins and the cysteine position in the amino acid sequence. Colors of the map illustrate a degree of oxidation: the red color indicates lower Cys_red_/Cys_ox_ ratio, depicting higher degree of oxidation. On the contrary, the blue color represents higher Cys_red_/Cys_ox_ ratio and therefore indicates lower oxidation state of the cysteine.

Next, we investigated in depth the redox state of the proteome of the failing hearts by analysing the oxidative state of cysteines residues, one of the main oxidative signaling mediators that are often modified (oxidized) upon reaction with RNOS [13,21]. To that end, we labeled reduced cysteines (Cys_red_) with a “light” N-ethylmaleimide (NEM; L), and subsequently reduced all oxidized cysteines (Cys_ox_). The newly formed thiol groups were labeled with “heavy” (d5)-NEM (H) prior proteomics analysis, enabling us to quantify the L/H (Cys_red_/Cys_ox_) ratio of 3767 cysteine-containing peptides across 25 samples. The dataset was then filtered to identify only differentially oxidized cysteines and, among them, selected peptides with reported Cys_red_/Cys_ox_ values in at least 70% of all samples. Setting these criteria enabled us to obtain a matrix of 659 cysteine peptides (Supplementary Table S2), on which Student *t*-tests were performed. A heatmap of 40 cysteine residues that were significantly more oxidized in failing hearts than in nonfailing samples is displayed in Figure 2. 

**Figure 3:**
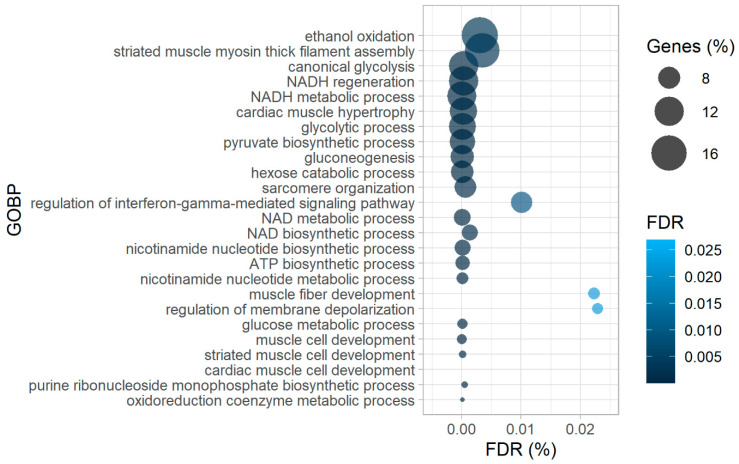
Gene-ontology-enrichment analysis of biological processes (GOBP) of corresponding proteins from significantly more oxidized cysteine-containing peptides in failing hearts as the input, revealing higher oxidation of proteins of contractile machinery and glucose metabolism. ATP—adenosine triphosphate, NAD(H)—nicotinamide adenine dinucleotide (reduced).

Amongst the proteins harboring more oxidized peptides in diseased hearts, many were enzymes involved in glucose metabolism, such as pyruvate kinase (PKM), phosphoglycerate mutase 1 (PGAM1), phosphoglucomutase 1 (PGM1), phosphoglycerate kinase 1 (PGK1), and mitochondrial aspartate aminotransferase (GOT2), which was corroborated by the gene ontology enrichment analysis of biological processes (GOBP) with more oxidized proteins as input (Figure 3). 

To corroborate our findings, we simulated different oxygen stress exposures in an in vitro cell system using commercially available human cardiomyocytes (AC16 cell line [22]). To this end, we cultured AC16 cells for 48 h either under physioxia (P; 5% oxygen) or normoxia (N; 21% oxygen). Additionally, cells cultured under physioxia were harvested either under physioxia (PP condition) or under normoxia (PN condition; to mimic sudden oxidative-stress exposure, as would happen in case of reperfusion injury) and then subjected to redox proteomic analysis. Interestingly, both prolonged (N) as well as short term (PN, up to 15 min) higher oxygen exposure of AC16 cells resulted in a higher oxidation (or at least a trend toward higher oxidation) of some of the same cysteine residues of the same glycolytic enzymes as in the failing hearts (Supplementary Figure S1A). Furthermore, among other cysteine residues that were significantly differentially oxidized between the three cell-culture conditions, several belong to proteins we observed as more oxidized in patients as well, such as neuroblast differentiation-associated protein AHNAK, PGK1, and filamin C (FLNC) (Supplementary Figure S1B). A detailed list of all L/H ratios of cysteine residues used for statistical analysis (202 for N versus PP, and 209 for PP versus PN) can be found in the Supplementary Table S3.1–2.

In patient samples we also detected higher oxidation of peptides matched to proteins responsible for the maintenance of contractile function in failing hearts (Figure 2), such as titin (TTN), filamin C (FLNC), LIM domain binding 3 (LDB3), and sarcoplasmic/endoplasmic reticulum calcium ATPase 2 (ATP2A2 or SERCA2). In support of these findings, the GOBP analysis of proteins more oxidized in failing hearts also resulted in enrichment of processes involved in muscle development and hypertrophy (Figure 3). Intriguingly, while redox regulation of titin has been proposed mainly for the I-band (where the spring region is located [23,24,25]), we found several significantly differentially oxidized sites from the A-band (repetitive fibronectin region), titin’s central hub for protein–protein interaction [25] in our patient dataset (Supplementary Table S4). However, it is noteworthy to mention that, although not significantly altered, we also detected a number of cysteine residues from the I-band which were more oxidized in failing hearts than controls (Supplementary Table S4).

Lastly, we also detected lower oxidation of eight cysteine-containing peptides in failing hearts (Supplementary Figure S2), some of which, belong to extracellular proteins involved in signaling, such as galectin-1 (LGALS1) as well as basement membrane-specific heparan sulphate proteoglycan core protein (Perlecan; HSPG2).

### 2.3. Protein Expression Patterns in Failing Hearts Demonstrate a Prominent Increase of Proteins Responsible for Extracellular-Matrix (ECM) Remodeling

**Figure 4:**
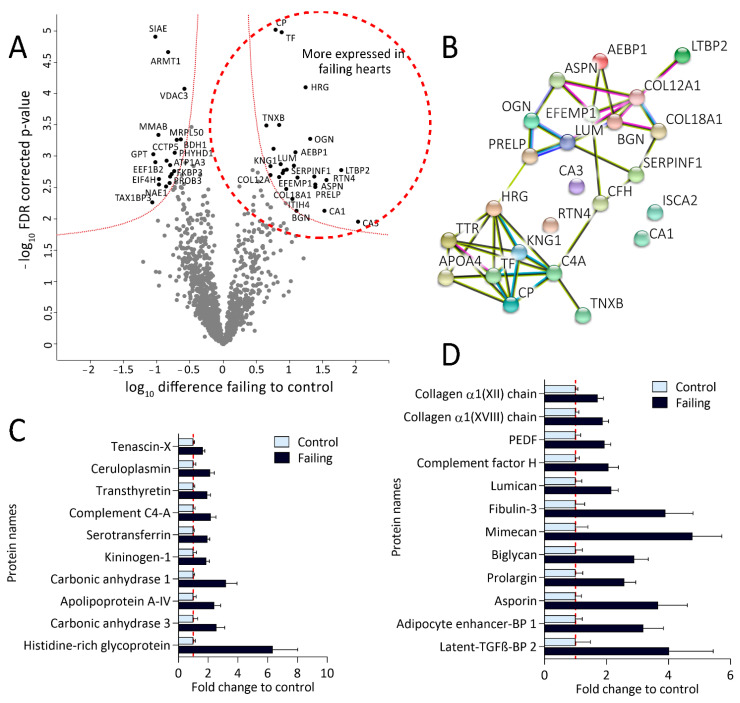
Proteomic analysis revealed altered protein expression patterns in failing hearts. (**A**): Volcano plot of proteins identified in failing and control hearts. Significant hits are labeled with their corresponding gene names and depicted in black. Proteins significantly more expressed in failing hearts are shown on the right while the proteins more abundant in the controls are located on the left side of the plot. (**B**): String protein-interaction analysis of proteins significantly more expressed in failing hearts results in two differential protein clusters (false discovery rate (FDR)-corrected *p*-value < 0.05, fold change (failing/control hearts) >1.5). (**C**): Bar plot displaying fold changes of significantly more expressed proteins from the lower String protein cluster (values normalized on the mean of the control group). (**D**): Bar plot representing fold changes of significantly up-regulated proteins from the upper String protein cluster, namely involved in extracellular matrix remodeling, wound healing, and fibrosis (values normalized on the mean of the control group). PEDF—pigment epithelium-derived factor (gene name: SERPINF1), BP—binding protein.

Protein abundance analysis of the sample dataset resulted in 2700 quantified proteins across 25 samples. Filtering for at least 80% valid values in at least one group reduced the matrix to 1500 proteins (Supplementary Table S5) on which Student *t*-tests were performed revealing 46 differentially expressed proteins between failing and nonfailing myocardium (Figure 4A, Supplementary Table S5). Interestingly, the ones significantly up-regulated in failing hearts (Figure 4A, right side of the volcano plot and Supplementary Table S5) clustered into two distinctive networks in the String protein-interaction analysis (Figure 4B). Their fold-changes in expression (compared to control) are shown in Figure 4C,D.

Proteins more abundant in failing heart tissue from the larger String protein cluster (Figure 4B,D) are mainly implicated in ECM remodeling and collagen assembly, as validated by the GOBP-enrichment analysis (Supplementary Figure S3). These proteins include several types of collagen, like alpha-1 XVIII and XII (COL12A1 and COL18A1, respectively), various small, leucine-rich proteoglycans, such as biglycan (BGN), asporin (ASPN), lumican (LUM), osteoglycin (OGN), proteotypical matricellular glycoprotein tenascin (TNXB) [26] as well as the newly discovered ECM protein Fibulin-3 (EFEMP1) [27]. In addition, we detected increased expression of a few proposed regulators of ECM remodeling (adipocyte enhancer-binding protein 1; AEBP1) and pigment epithelium-derived factor; SERPINF1) [28,29,30], as well as transforming growth-factor beta (TGF-β) targets and interaction partners (including latent-transforming growth-factor beta-binding protein 2; LTBP 2) [31,32,33]. Finally, also previously proposed diagnostic markers of heart failure, including carbonic anhydrase 1 (CA1) [34] and ceruloplasmin (CP), were among the more abundant proteins in failing hearts [35]. 

**Figure 5:**
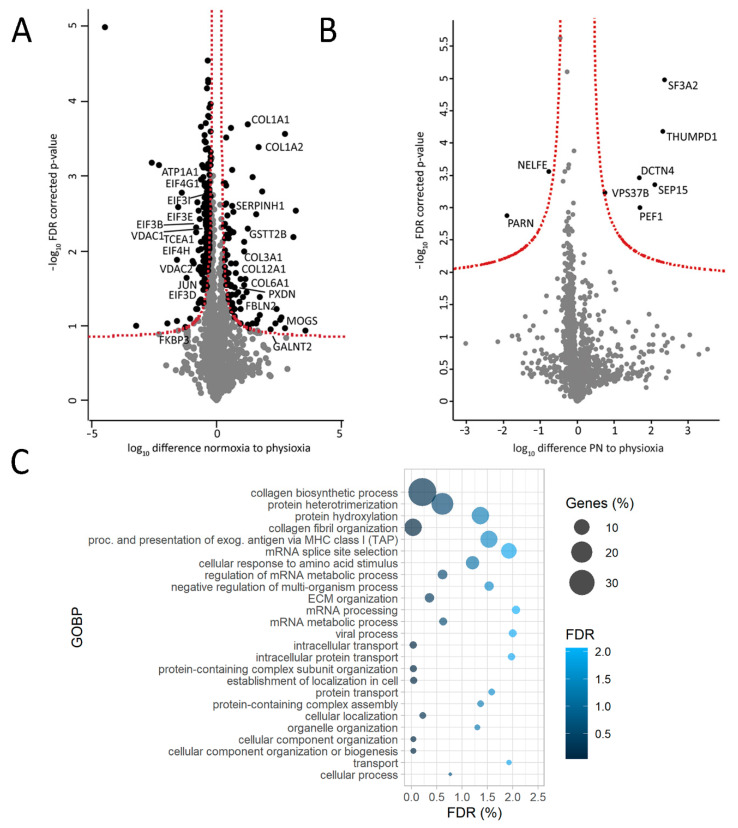
AC16 cells cultured under higher oxygen concentration (higher oxidative stress) display some similar protein signatures as failing human hearts. (**A**). Volcano plot of differentially expressed proteins between normoxic (N) and physioxic conditions (harvested under physioxia, PP); (**B**). Volcano plot of differentially expressed proteins between cells cultured in physioxia but harvested in normoxia (PN) and cells cultured and harvested under physioxia (PP). (**C**). Gene-ontology enrichment of biological process (GOBP) with significantly more abundant proteins in normoxia (N) compared to physioxia (PP) (proteins used for input must have passed the significance threshold of false discovery rate (FDR)-corrected *p*-value < 0.05 and have a fold change (compared to physioxia) >1.3). N = 3 per condition, FDR.

Interestingly, changes in ECM were also observed in in vitro cultured AC16 cells. Culturing cells in higher oxygen environment (21%; normoxia; N) compared to physioxia (5%; P) led to a prominent increase in the production of various types of collagen (Figure 5A) and seemed to influence ECM organization as corroborated by the GOBP analysis with proteins higher abundant in N as input (Figure 5C). This was not the case upon short term oxygen stress exposure (harvesting of cells cultured in physioxia under normoxia; PN condition; Figure 5B). A list of AC16 proteins harvested under different conditions used for statistical analysis (1751 proteins for both N versus PP as well as PN versus PP) can be found in Supplementary Tables S3.3–4.

### 2.4. Reduced Expression of Ion Regulators and Members of Translation and Protein-Folding Machinery in Failing Hearts 

**Figure 6:**
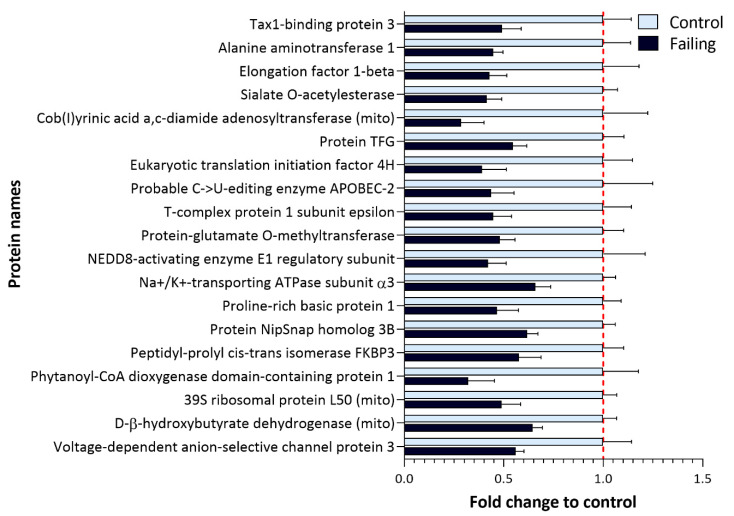
Proteins with a significantly lower expression in failing hearts compared to nonfailing control samples. Bar plot representing fold change in the expression of significantly less expressed proteins in failing hearts (Figure 4A, left side of the volcano plot) which include several metabolic enzymes as well as proteins implicated in protein translation and ion transport (FDR-corrected *p*-value < 0.05, fold change (failing/control hearts) <0.7).

Failing hearts display reduced expression of several proteins (Figure 4A, left side of the volcano plot) including potentially relevant diagnostic candidates. Their fold changes (compared to control) hearts are shown in Figure 6. 

The expression of two important ion regulators, namely ATPase α3 (ATP1A3) and voltage-dependent anion-selective channel 3 (VDAC3), was found to be down-regulated in failing hearts. This is in line with previous reports suggesting about 40% reduction in Na^+^/K^+^ ATPase abundance in failing hearts [36] (Supplementary Table S5). 

Next to regulators of ion homeostasis, we also detected down-regulation of proteins involved in processes of translation, such as elongation factor beta and translation-initiation factor 4H, as well as down-regulation of proteins responsible for maintaining proper protein folding, such as NEDD8-activating enzyme, for which a protective role against apoptosis has been proposed in cardiomyocytes [37]. Furthermore, a member of FK506-binding proteins (FKBP, prolyl isomerases) FKBP3 (also known as FKBP25) was found to be down-regulated in failing hearts. In addition to acting as a chaperone in proper protein folding [38], FKBP3 was implicated in the maintenance of the Ca^2+^ homeostasis [39], and FKBP3 overexpression was reported to protect cells from oxygen and glucose deprivation injury [40]. 

It is noteworthy to mention that similar trends were observed in AC16 cells. In normoxia, various translation-initiation factors (Figure 5A), as well as several ion channels including ATPase α1 (ATP1A1) and voltage-dependent anion-selective channels 1 and 2 (VDAC1 and 2), were found to be reduced (Figure 5A). In addition, FKBP3 (FKBP25) showed a trend of being down-regulated in normoxic AC16 cells (Figure 5A). This was not the case upon short-term higher oxygen exposure (Figure 5B). A list of quantified proteins from AC16 cells can be found in Supplementary Tables S3.3–4.

## 3. Discussion

Oxidative stress is an important contributor to the pathophysiology of heart failure [41]. In this study, we demonstrated that LV tissue from failing hearts due to ICM or DCM is indeed exposed to higher levels of oxidative stress, manifested through reduced GSH/GSSG ratios. These findings are in line with previous reports of lower GSH values in blood of DCM patients and reduced GSH/GSSG ratios in hearts of patients and animal models with cardiomyopathy [11,42]. On the same note, glutathione was also reported to be severely depleted in coronary patients with reduced left ventricular ejection fraction (EF < 40–45 %) in atrial tissue collected during cardiac surgeries [7].

Next to perturbations of glutathione homeostasis, we also addressed the cysteine redox proteome and observed that failing hearts depict higher oxidation status of various proteins, including a number of glucose metabolic enzymes. Previous studies demonstrated that elevated glycolytic flux is a hallmark of the failing heart [43,44]. Insufficient oxygen supply during cardiac ischemia was reported to shift metabolism from fatty-acid oxidation toward glycolysis to fuel ATP production [43,44], which was not reversed even upon restoring oxygen levels [43]. Although it is still largely unknown how this affects the functional status of the enzymes involved, it seems that RNOS can directly modulate and may even inhibit the activity of glycolytic enzymes in other tissues [45]. Moreover, changes in the oxidative state of metabolic enzymes can also be a result of RNOS-driven deregulation of antioxidative enzymes. For example, PGAM (including the Cys^153^ detected in this study (Figure 2)) was recently described as a glutaredoxin (Grx1) target in hepatic cells and as such, could be involved in redox signaling [46]. In some cases, higher oxidation of cysteine residues of an enzyme might even serve as defence mechanism to prevent its denaturation upon oxidative-stress exposure. This was proposed for PKM, which can form interprotein disulfide bridges (involving detected Cys^49^), leading to enzyme dimerization that can prevent oxidation damage [47]. Interestingly, when culturing or harvesting AC16 cells were under normoxia (i.e., higher oxygen stress compared to physioxia), exactly these cysteine residues (Cys^49^ of PKM, Cys^153^ of PGAM) displayed a trend toward higher oxidation, indeed suggesting their sensitivity to oxidative-stress perturbations (Figure S1A). It is noteworthy to mention that higher oxidation of glucose metabolic enzymes (including Cys^49^ of PKM) was also reported for cardiac tissue of mice fed a Western diet [48], which might suggest that other contributors, such as nutrition, can also influence the redox state of the cardiac proteome. In this study, we did not observe a significant difference in the body mass index (BMI) between the failing heart and control patients (BMI 25 ± 8 and 27 ± 4, for failing and control group, respectively) which would imply prominent lifestyle discrepancies. However, we do know that the patients which underwent heart transplantation were often suffering from hypertension and/or coronary artery disease. Therefore, it cannot be excluded that some of the observed phenotypes are actually a consequence of different life habits between the two investigated groups. 

In addition to glycolytic enzymes, failing hearts also displayed higher oxidation of cysteine residues of several constituents of the contractile machinery, including titin, a giant scaffold protein which acts as a molecular spring and represents a central node of mechanic signaling [49]. Oxidation of critical myofilament proteins has been recognized as one of the causes leading to contractile dysfunction under oxidative stress [13,50,51]. In particular, higher oxidation of titin in failing hearts is in agreement with previous reports suggesting that oxidation of titin through the formation of disulfide bonds contributes to increased passive stiffness in failing hearts [52]. However, in addition to the reported “oxidation-prone” cysteine residues from titin’s elastic I-band [23], we herein report several more oxidized peptides from the A-band, where the majority of protein–protein interactions take place [25]. 

Our redox dataset was additionally complemented with quantitative proteomics, which revealed a panel of up-regulated ECM proteins in failing human left-ventricular heart tissues. ECM remodeling is a key feature of heart failure [53], and failing hearts undergo significant structural remodeling toward irreversible fibrosis [54]. As such, ECM remodeling represents an attractive target for early diagnostics and treatment; however, a more detailed understanding of molecular events driving ECM remodeling is needed as a prerequisite for translation to clinical utility [54,55]. Several ECM proteins detected in this study have already been proposed as potential markers of heart failure, including osteoglycan (mimecan) [56], fibulin-3 [57], biglycan [58,59], and asporin [60]. Our data further stratifies the usefulness of ECM proteins as potential biomarkers and emphasizes the need for further research in this direction. It is also noteworthy to mention that culturing AC16 cardiomyocytes under higher-oxidative-stress conditions (in normoxia compared to physioxia) also led to collagen production and reorganization of ECM (Figure 5A,C), suggesting that oxidative stress could be one of the drivers of ECM remodeling.

Contrary to higher expression of ECM proteins, we found reduced levels of several proteins involved in ion homeostasis, including ATPase α3 (ATP1A3), for which a 40% lower expression was already reported in failing human hearts [36]. Interestingly, proton accumulation and RNOS signaling through cysteine modifications in ischemic cardiomyocytes reportedly inhibit the activity of these pumps [43,61]. Accordingly, in failing hearts we also detected higher oxidation of another Na^+^/K^+^ ATPase isoform, namely ATPase 2 (ATP2A1/2; Figure 2), suggesting not only reduced abundance but also potentially redox-dependent changes in activity of these ion-handling proteins. Similarly, normoxic AC16 cells displayed a reduction in abundance of other important ion channels such as ATPase α1, as well as VDAC2 and 3 as compared to physioxia (Figure 5A,C, Supplementary Tables S3.3–4), corroborating that oxidative stress might modulate the expression of these proteins. 

Lastly, while we identified a trend toward less protein translation in hearts from patients suffering from ischemic or dilated cardiomyopathy (Figure 4A) and in AC16 cells cultured in normoxia compared to physioxia (Figure 5A,C), very recent reports suggest that higher translation rates correlate with pressure overload and development of hypertrophy [62,63]. In the heart, the mammalian target of rapamycin complexes 1 and 2 (mTORC1 and 2) are the master regulators of cardiac translation-activating protein synthesis as a response to injury or pressure overload to enable adaptive hypertrophy [63], and hypertrophic signals were shown to activate mTORC and boost protein synthesis [64]. However, prolonged exposure to other stressors can halt cardiac protein synthesis by inactivating mTORC1 (via tuberous sclerosis 1 (TSC1/2) or inhibition of Rheb) [63,64,65]. Interestingly, Zhang et al. [66] showed that inhibition of mTORC1 in mice (through heart-specific gene ablation) led to inhibition of cardiac translation, inadequate cardiac remodeling, and eventually resulted in lethal dilated cardiomyopathy. Data from our study complement these reports suggesting that the rate of translation can act as critical discriminators of different HF etiologies.

## 4. Materials and Methods

If not stated otherwise, all chemicals were purchased from Sigma-Aldrich (St. Louis, MO, USA).

### 4.1. Tissue Specimens 

LV myocardial tissue samples were obtained from end-stage failing heart explants (*n* = 15 males, mean age = 63 ± 4 years, mean body mass index (BMI) = 25 ± 8, mean left-ventricular ejection fraction (EF) = 30 ± 11%, body mass index () and nonfailing human control hearts (*n* = 10 males, mean age = 63 ± 3 years, mean BMI = 27 ± 4, EF = 63 ± 4.3%) that were not accepted for transplantation. Fifteen end-stage failing heart explants included three patients suffering from end-stage DCM (*n* = 3 males, mean age 63 ± 3.9 years, EF 22 ± 5.8%) and 12 patients with ICM (*n* = 12 males, mean age 63 ± 5 years, EF 33 ± 11%). All hearts were stored in the same commercially available cardioplegic solution upon collection, then sampled, snap-frozen in liquid nitrogen, and stored at −80 °C. 

### 4.2. Study Approval and Ethical Aspects

The use of human biomaterials was approved by the ethics committee of the Medical University of Graz (20-277 ex 08/09, 26-355 ex 13/14, 28-508 ex 15/16) and conformed with all pertaining regulations and the principles of the Declaration of Helsinki [67].

### 4.3. Cell Culture 

AC16 cardiomyocytes (SCC109) were cultured at 37 °C in phenol-red free DMEM/F12K medium (D6434), supplemented with 12.5% FBS (Gibco, Thermo Fisher, Waltham, MA, USA), penicillin-streptomycin, and 2 mM glutamine. One day prior to experiment, 60.000 cells were seeded in quadruplicates per condition in 12 well plates and on the following day either moved to a physioxia incubator (5% oxygen) or were left at a regular incubator (21% oxygen). Cells were then harvested after 48 h either under normoxia (N and PN conditions) or under physioxia (PP condition) and prepared for proteomic analysis. In case of the PN condition, the total time between the removal of cells from the physioxia chamber and cell lysis was up to 15 min.

### 4.4. Glutathione Measurement Sample Preparation

Samples for glutathione analysis were prepared as previously described [17]. In brief, 4–7 mg of the tissue was incubated in 2.5 mM N-ethylmaleimide (light NEM) in phosphate buffer saline (PBS) for 20 min at room temperature (RT) to alkylate free thiols (reduced cysteines). Proteins were precipitated with cold 80% methanol spiked with internal standard (IS, ^13^C_2_, and ^15^N-GSH-d5-NEM) for 20 min at −20 °C. Samples were centrifuged to pellet the protein; polar supernatants were transferred to a new tube and dried down under a stream of N_2_. Dried samples were reconstituted in 100 µL of 50 mM ammonium acetate (AA) and extracted with dichloromethane to remove excess of NEM. Consequently, samples were reduced by adding 2.5 µL of 50 mM tris(2-carboxyethyl) phosphine (TCEP) in AA to 45 µL of NEM-free polar phase and incubated for 30 min at 37 °C to reduce disulfides. Lastly, for the second alkylation step, 2.5 µL of 100 mM of d5-NEM/AA was added (as heavy label for the originally oxidized cysteine residues), and the samples were incubated for another 20 min at RT and diluted 1:5 prior to LC-MS/MS analysis. Detailed LC-MS/MS parameters are described in the Supplement.

### 4.5. Proteomics Sample Preparation

Protein pellets from glutathione sample preparation were dissolved in 200 µL of 100 mM Tris pH = 8.5, containing 1% sodium dodecyl sulphate (SDS) and 10 mM NEM, sonicated 4 × 10 s at 80% amplitude (Bandelin, Berlin, Germany) and further lysed for 30–60 s with an Miccra D-1 tissue homogenizer (Donau Lab, Kyev, Ukraine). Tissue lysates were then centrifuged, and 100 µg of protein was acetone-precipitated overnight. Protein pellets were redissolved in 87.5 µL of 50% trifluoroethanol (TFE) in 50 mM ammonium-bicarbonate (ABC) and reduced with TCEP (5 mM final concentration) for 20 min at 60 °C. Reduced samples were subjected to a second alkylation step using d5-NEM (10 mM final concentration, 20 min at RT) to label oxidized cysteines and diluted 1:4 with 25 mM ABC prior to digestion with trypsin overnight at 37 °C. In addition, 40 µg of each tryptic digest was consequently fractionated with the Pierce high pH reversed-phase peptide fractionation kit (Thermo Fisher, Waltham, MA, USA) according to manufacturer’s instructions into eight fractions. After fractionation, fraction one and seven and fraction two and eight were combined (F1 + F7 and F2 + F8, respectively). Fractions were dried down and resuspended in 16 µL (32 µL for combined fractions) of running buffer A (5% acetonitrile, 0.1% formic acid) and subjected to LC-MS/MS analysis.

Cell culture samples were harvested in 100 µL of lysis buffer, no fractionation was carried out, and samples were desalted offline prior to LC-MS/MS analysis. In addition, 500 ng protein per each sample was used for injection. 

### 4.6. LC-MS/MS Parameters (Proteomics)

Redox and label-free proteomics: Chromatography was carried out on an Ultimate 3000 RCS Nano Dionex system equipped with an Ionopticks Aurora Series UHPLC C18 column (250 mm × 75 µm, 1.6 µm) (Ionopticks, Fitzroy, Australia). Total LC-MS/MS run per each fraction/run was 133 min with the following gradient (solvent A is 0.1% formic acid in water; solvent B is acetonitrile containing 0.1% formic acid): 0–18 min: 2% B; 18–100 min: 2–25% B; 100–107 min: 25–35% B, 107–108 min: 35–95% B, 108–118 min: 95% B; 118–118.1 min: 95–2% B; and 118.1–133 min: 2% B at a flow rate of 300 nL/min and 50 °C. The maXis II ETD mass spectrometer (Bruker, Billerica, MA, USA) was operated with the captive source in positive mode employing the following settings: mass range: 200–2000 m/z, 2 Hz, capillary 1600 V, dry gas flow 3 L/min at 150 °C, nanoBooster 0.2 bar, precursor acquisition control set to fragment the top 20 most abundant peaks. The mass spectrometry proteomics datasets were deposited to the ProteomeXchange Consortium via the PRIDE partner repository [68] with the dataset identifiers: Patient samples: PXD021261 (Reviewer account details: Username: reviewer43721@ebi.ac.uk; Password: VQlOMIWk); cel- culture samples: PXD023620 (Reviewer account details: Username: reviewer_pxd023620@ebi.ac.uk, Password: yupaRKpx).

### 4.7. Proteomics Data Processing

#### 4.7.1. Patient Samples

Data analysis including database search, light-to-heavy (L/H) ratio calculation. as well as label-free protein quantification (LFQ), was performed using MaxQuant (v1.6.1.0) [69,70,71]. False discovery rate (FDR) for database matching was set to 1% and minimum peptide length to six amino acids. Match between run feature was enabled with the match and alignment windows of 3 and 20 min, respectively. As samples were measured in two separate batches, LFQ dataset was further normalized by being first log transformed (base 2), then mean centred within each batch [72,73] and finally merged back into one dataset prior statistical analysis. 

Redox proteomics: For peptide L/H (light to heavy, NEM to d5-NEM) ratio estimation, NEM and d5-NEM were configured as a “light” (NEM) and “heavy” (d5-NEM) label pair in MaxQuant. Methionine oxidation was selected as dynamic modification and no static modifications were defined. Statistical analysis was performed in Perseus (v1.6.5.0) [74]. A MaxQuant table containing peptide specific L and H intensity values for each sample was imported into Perseus, and the matrix was log_2_ transformed. Consequently, L/H ratios were calculated per sample (log_2_(Intensity L)-log_2_(Intensity H)) and the matrix was filtered to keep only cysteine-containing peptides and exclude all contaminants. The matrix was further filtered to contain only those peptides with reported L/H ratios in at least 70% of all samples. Missing values were imputed from normal distribution (sampling around zero value; 0.2, 0.5 width, and downshift, respectively) and a two-tailed Student *t*-test was performed between failing and nonfailing sample groups (without multitesting correction). Only hits with a *p*-value below 0.05 and a fold change of the ratio (compared to failing hearts) of more than 1.35 were considered significant. 

Label-free proteomics: For LFQ analysis, methionine oxidation, NEM and d5-NEM cysteine modifications were selected as dynamic modifications, and no static modifications were defined. At least two peptides were required for quantification. In Perseus, the table with protein LFQ intensities was as well filtered for contaminants, after which the matrix was further filtered to contain at least 80% valid values in at least one of the groups. Missing values were imputed from a normal distribution (width 0.3, downshift 1.8) and two-sample *t*-tests corrected for multitesting were performed between the groups (FDR 5%, S0 0.1). For String analysis of LFQ data, significantly changed proteins between the conditions (*p*-value of less than 0.05 and a fold change of more or less than 1.5 (compared to the control)) were used as input. 

#### 4.7.2. Cell Culture Samples

Cell-culture proteomics data were processed using Max Quant version 1.6.17.0 and Perseus 1.6.5.0. Statistical analysis was carried out with three replicates per condition. Redox proteomics analysis was performed the same way as for the tissue samples, with the only difference that prior statistical testing the matrix was filtered to keep only those cysteine residues with valid L/H ratios in all samples (100% valid values). Student *t*-test *p*-value < 0.05 was taken as significance threshold. Label-free proteomics data analysis was also carried out as described, with small adjustments: Prior to statistical testing, the matrix was filtered to keep only those proteins with reported valid values in at least three samples in at least one group. Missing values were then imputed from a normal distribution with a width of 0.2 and a downshift of 2. Student t-tests were performed as described for the tissue samples (FDR corrected *p*-value < 0.05, S0 0.01). For String analysis of LFQ data, significantly more abundant proteins in the normoxic group (Student *t*-test *p*-value of less than 0.05 and a fold change more than 1.3 (compared to the PP)) were used as input.

### 4.8. Enrichment and Protein–Protein Interaction Analysis

Gene-ontology analysis was performed using the STRING online database (v11.0, https://string-db.org/, access on 6 July 2020). For enrichment analysis, the threshold for FDR-corrected *p*-value was 0.05. For protein–protein interaction analysis a medium confidence of 0.4 was allowed and interactions based on evidence were displayed. Limits in the number of connectors were not set.

### 4.9. Statistics

Data is reported as mean values ± standard error of mean (S.E.M.). For significance testing, an unpaired Student *t*-test was performed with a *p*-value of 0.05 as the significance threshold.

## 5. Conclusions

Here we applied a novel, combined MS-based approach to address global and peptide specific differential oxidation and differential protein expression of human failing hearts. Using the same tissue explants for the analysis of cysteine redox proteome, as well as glutathione status and quantitative proteomics enabled us to carry out precise and to our knowledge most comprehensive redox and proteomics analysis of the human failing heart tissue. As a result of this work, we report decreased reduced to oxidized glutathione ratios in failing hearts, as well as increased oxidation of specific proteins including glycolytic enzymes and members of the myocyte contractile machinery. Failing hearts also displayed changes in expression of many proteins involved in ECM remodeling, ion homeostasis, metabolism, and protein translation, all of which may act in concert and contribute to the progression of heart failure. Strikingly, similar observations could be made in an in vitro system of cardiomyocytes cultured under different oxygen concentrations suggesting a major role of local oxygen concentrations in proteome remodeling. Altogether, we used state-of-the-art MS-based methodology to provide in-depth analyses of proteome and redox changes which occur in correlation with HF, laying a rich basis for further prognostic and mechanistic investigations of relevant proteins and their associated redox changes.

## Data Availability

The mass spectrometry proteomics datasets have been deposited to the ProteomeXchange Consortium via the PRIDE partner repository [68] with the dataset identifiers: Patient samples: PXD021261 (Reviewer account details: Username: reviewer43721@ebi.ac.uk; Password: VQlOMIWk); cell culture samples: PXD023620 (Reviewer account details: Username: reviewer_pxd023620@ebi.ac.uk, Password: yupaRKpx).

## Supplementary Materials

The following are available online at https://www.mdpi.com/1422-0067/22/4/1787/s1, Supplement_Tomin et al._MS-BASED REDOX AND PROTEIN PROFILING OF FAILING HUMAN HEARTS.docx. File containing supplementary methods, Supplementary Table S1 and Supplementary Figures S1–S3. Supplementary Table S2. Cysred to Cysox ratios of peptides from redox analysis (patients samples).xlsx. Table of all cysteine peptides from patient samples used for statistical analysis with corresponding Cys_red_/Cys_ox_ (L/H) ratios. Supplementary Table S3(1–4). Proteomics data AC16 cells (redox and quantitative proteomics).xlsx. S3.1. Table of all cysteine peptides from AC16 cells used for statistical analysis with corresponding Cys_red_/Cys_ox_ (L/H) ratios (PP *versus* N condition). S3.2. Table of all cysteine peptides from AC16 cells used for statistical analysis with corresponding Cys_red_/Cys_ox_ (L/H) ratios (PP *versus* PN condition). S3.3. Table of all quantified proteins in AC16 cells used for for statistical analysis (PP *versus* N condition). S3.4. Table of all quantified proteins in AC16 cells used for for statistical analysis (PP *versus* PN condition). Supplementary Table S4. Cysred to Cysox ratios of titin peptides.xlsx. Table of all cysteine-containing titin peptides from patient samples used for statistical analysis with corresponding Cys_red_/Cys_ox_ (L/H) ratios. Supplementary Table S5. Quantitative proteomics (patients samples).xlsx. Table of all quantified proteins in patient samples used for for statistical analysis (control versus failing hearts).
