# Supplementary material for: Mass Spectrometry-Based Redox and Protein Profiling of Failing Human Hearts
Source: Int J Mol Sci. 2021 Feb 11;22(4):1787. doi: 10.3390/ijms22041787 (PMC7916846; doi:10.3390/ijms22041787)
Supplement: Supplementary file 1 [file ijms-22-01787-s001.zip › Supplement_Tomin et al_MS-BASED REDOX AND PROTEIN PROFILING OF FAILING HUMAN HEARTS.pdf]

MASS SPECTROMETRY-BASED REDOX AND PROTEIN PROFILING OF FAILING HUMAN HEARTS

Tamara Tomin[1,2,3], Matthias Schittmayer[1,2,3#], Simon Sedej[3,4, 5], Heiko Bugger[4], Johannes Gollmer[4], Sophie Honeder[2,3], Barbara Darnhofer[2,3], Laura Liesinger[2,3], Andreas Zuckermann[6], Peter P. Rainer[3,4#$] and Ruth Birner-Gruenberger[1,2,3#$]

[1] Institute of Chemical Technologies and Analytics, Faculty of Technical Chemistry, Vienna University of Technology - TU Wien, Getreidemarkt 9/164, 1060 Vienna, Austria

[2] Diagnostic and Research Institute of Pathology, Medical University of Graz, Stiftingtalstrasse 6, 8010 Graz, Austria

[3] BiotechMed-Graz, Mozartgasse 12/II, 8010 Graz, Austria

[4] Division of Cardiology, Medical University of Graz, Auenbruggerplatz 15, 8036 Graz, Austria

[5] Faculty of Medicine, University of Maribor, Maribor, Slovenia

[6] Cardiac Transplantation, Department of Cardiac Surgery, Medical University of Vienna, Spitalgasse 23, 1090 Vienna, Austria

[$] Authors contributed equally and thus should be considered co-last authors

[#] Corresponding authors: Peter P. Rainer, Division of Cardiology, Medical University of Graz, Auenbruggerplatz 15, 8036 Graz, Austria, and Ruth Birner-Gruenberger and Matthias Schittmayer, Institute of Chemical Technologies and Analytics, Faculty of Technical Chemistry, Vienna University of Technology - TU Wien, Getreidemarkt 9/164, 1060 Vienna, Austria, emails: peter.rainer@medunigraz.at, ruth.birner-gruenberger@tuwien.ac.at and matthias.schittmayer@tuwien.ac.at

SUPPLEMENTARY MATERIAL AND METHODS

If not stated otherwise, all chemicals were purchased from Sigma Aldrich.

LC-MS/MS parameters (Glutathione measurements)

Glutathione was measured as described [1]. Briefly, chromatography was carried out on a Dionex UltiMate 3000 system equipped with a Zorbax SB-C18 column (50 mm x 4.6 mm, 1.8 µm, Agilent, USA). The following gradient employing solvent A (0.1 % formic acid in water) and solvent B (0.1 % formic acid in acetonitrile) at a flow rate of 0.3 ml/min was applied: 0 – 10 min 1 – 30 % B, 10 – 15 min 30 – 70 % B, 15 – 20 min 1 % B. Injection volume was 10 µl. The ABSciex 4000 QTRAP mass spectrometer (as published [1]) or a TSQ Access Max triple quadrupole (Thermo Scientific, USA) equipped with electrospray ionization (ESI) source was used as detector, operating in positive MRM mode. TSQ parameters were the following: spray voltage 3000 V, capillary temperature 240 °C, vaporizer temperature 300 °C, and sheath gas, ion sweep gas and aux gas pressures were 35, 0 and 5 units respectively. Tube lens offset was set to 48 for the lower molar masses and skimmer offset was set to 0. A list of transitions with their corresponding collision energies for TSQ is shown in Suppl. Table 1.

Supplementary Table S1. Multiple reaction monitoring (MRM) table of transitions for glutathione and glutathione derivatives used for measurements on TSQ Access Max triple quadrupole. Transitions highlighted in bold were used for quantitation. All parents and products were measured as $[M+H]^+$ ions.

Metabolite	Parent ion	Product ion	Collision energy
**GSH**	**308**	**179**	**19**
**GSH-NEM (Transition 1)**	**433.16**	**201**	**22**
GSH-NEM (Transition 2)	433.16	304	15
**GSH-d5-NEM (Transition 1)**	**438.16**	**206**	**22**
GSH-d5-NEM (Transition 2)	438.16	309	15
**$^{13}C_2$, ^{15}N-GSH-d5-NEM (IS)**	**441.16**	**206**	**22**
**GSSG**	**613.2**	**355.3**	**10**

SUPPLEMENTAL FIGURES

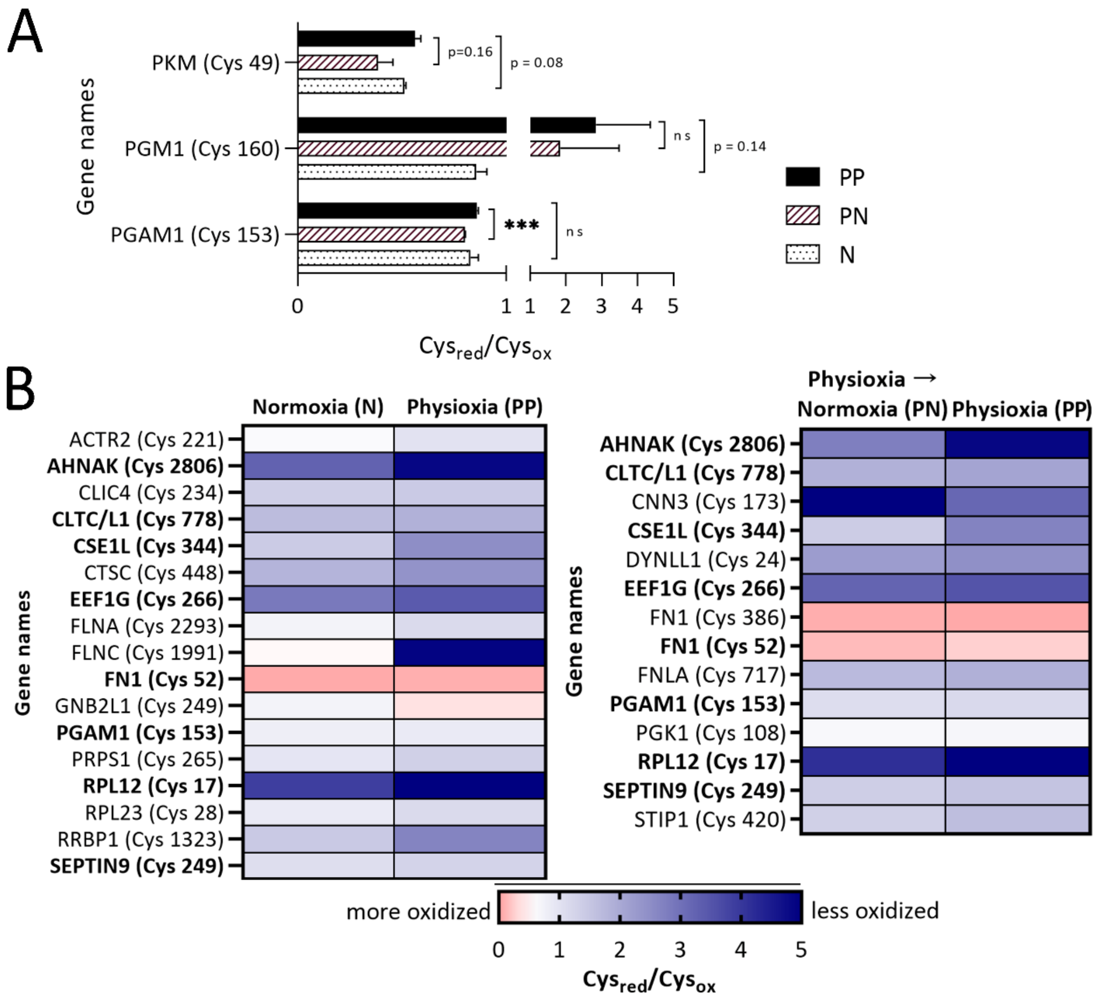

Figure S1. AC16 cells cultured under higher oxygen concentration (normoxia) compared to physioxia. A. Reduced to oxidized ratios (Cys_{red}/Cys_{ox}) of cysteine residues belonging to three glycolytic enzymes found significantly more oxidized in failing heart samples. N = 3 per condition, *** Student t-test p-value < 0.001, ns – not significant.

B. Heatmap of significantly more or less oxidized cysteine residues under different culture (left) and harvesting conditions (right). Same residues that were differentially oxidized in both N and PN conditions compared to PP are marked in bold. Colours of the map illustrate the oxidation state: red colour depicts a lower Cys_{red}/Cys_{ox} ratio resembling a higher degree of oxidation. On contrary, blue colour depicts a higher Cys_{red}/Cys_{ox} ratio and therefore resembling a lower oxidation state of the cysteine.

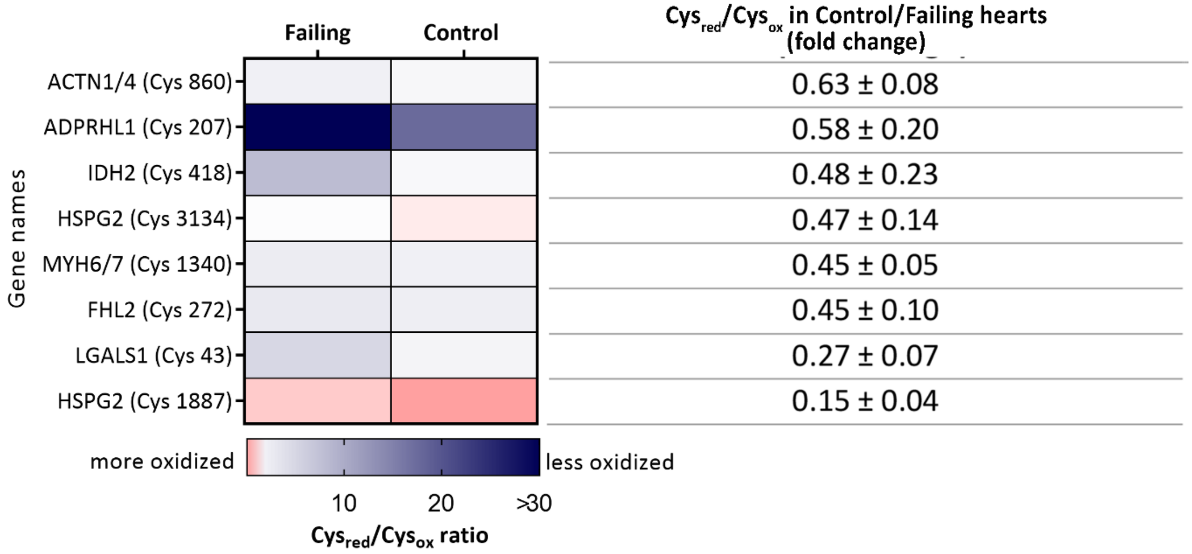

Cys_{red}/Cys_{ox} in Control/Failing hearts (fold change)
0.63 ± 0.08
0.58 ± 0.20
0.48 ± 0.23
0.47 ± 0.14
0.45 ± 0.05
0.45 ± 0.10
0.27 ± 0.07
0.15 ± 0.04

Figure S2. Less oxidized peptides in failing hearts. A: Significantly less oxidized (p-value < 0.05, fold change (Cys_{red}/Cys_{ox} ratio of control/failing heart < 0.75) cysteine containing peptides in failing hearts are labelled with gene names of their corresponding proteins and cysteine position in the amino acid sequence. Colours of the map illustrate the oxidation state: red colour suggests lower Cys_{red}/Cys_{ox} ratio meaning higher degree of oxidation. On contrary, blue colour represents higher Cys_{red}/Cys_{ox} ratio and therefore lower oxidation state of the cysteine.

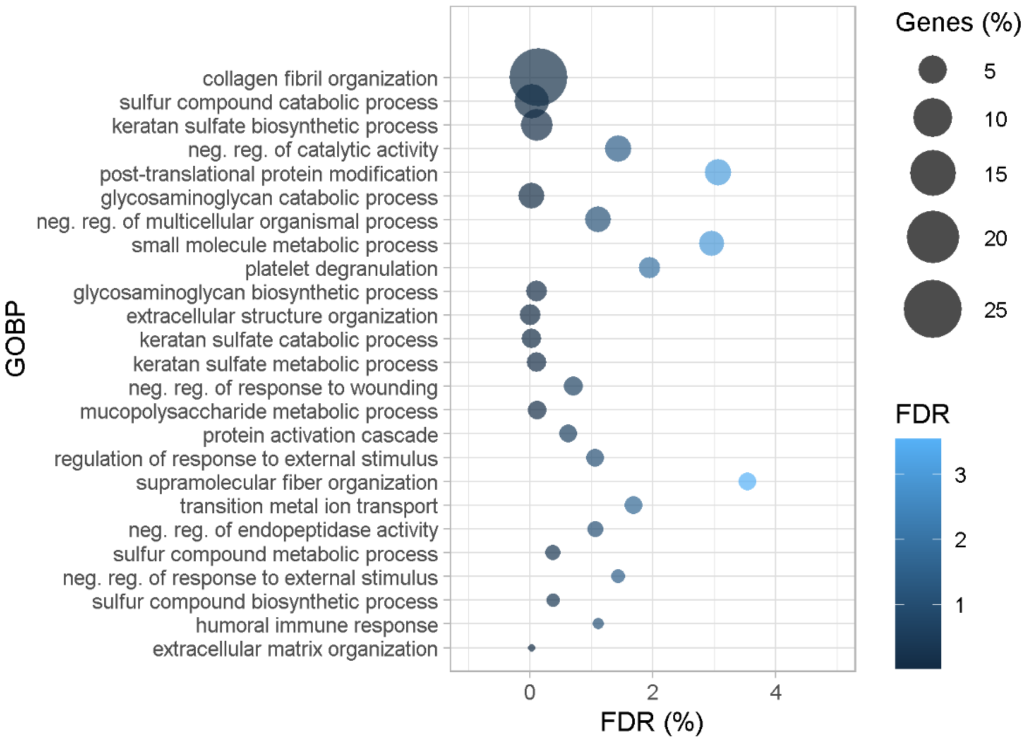

Figure S3. GOBP enrichment analysis of proteins significantly more expressed in failing hearts reveals a high upregulation of pathways involved in extracellular matrix remodeling and fibril organization.

References

1. Tomin, T.; Schittmayer, M.; Birner-Gruenberger, R. Addressing Glutathione Redox Status in Clinical Samples by Two-Step Alkylation with N-ethylmaleimide Isotopologues. *Metabolites* **2020**, *10*, 71, doi:10.3390/metabo10020071.
